# Microwave-assisted hydrotropic pretreatment as a new and highly efficient way to cellulosic ethanol production from maize distillery stillage

**DOI:** 10.1007/s00253-021-11258-2

**Published:** 2021-04-09

**Authors:** Mikulski Dawid, Kłosowski Grzegorz

**Affiliations:** Department of Biotechnology, Kazimierz Wielki University, ul. K. J. Poniatowskiego 12, 85-671 Bydgoszcz, Poland

**Keywords:** Microwave-assisted hydrotropic pretreatment, Distillery stillage, Cellulosic ethanol, Sodium cumene sulfonate

## Abstract

**Abstract:**

Aim of the study was to assess the suitability of the combined use of microwave radiation and sodium cumene sulfonate under optimized process conditions for the preparation of maize stillage biomass as a raw material for the production of cellulosic ethanol. The key parameter guaranteeing a high level of lignin removal from biomass (ca. 44%) was concentration of hydrotrope. Even at high biomass concentration (16% w/v) and a cellulase enzyme dose of about 4 filter-paper units/g, maize stillage biomass subjected to microwave-assisted hydrotropic pretreatment was highly susceptible to enzymatic degradation, which resulted in 80% hydrolysis yield. It is possible to obtain a fermentation medium with a very high glucose concentration (up to 80 g/L), without fermentation inhibitors and, as a consequence, to reach a very high level of sugar conversion to ethanol (concentration above 40 g/L), even as much as 95% of theoretical yield. Microwave hydrotropic treatment with sodium cumene sulfonate is a very effective way to prepare waste maize stillage biomass for the production of cellulosic ethanol. The degradation of the lignocellulose structure by the simultaneous use of microwaves and hydrotropes ensured a high degree of conversion of structural polysaccharides to bioethanol. The method provides a high level of enzymatic degradation of cellulose, leading to a medium with high content of released sugars suitable for bioconversion, which is in line with assumptions of the second-generation ethanol production technology.

**Key points:**

• *Microwave-assisted hydrotropic pretreatment is a new way to cellulosic ethanol production.*

• *Microwave-assisted hydrotropic delignification removes 44% of lignin from biomass.*

• *No fermentation inhibitors are obtained after microwave-assisted hydrotropic pretreatment.*

• *High ethanol concentration (above 40 g/L) and fermentation yield (95% of theoretical yield) from biomass after microwave-assisted hydrotropic pretreatment.*

## Introduction

The second-generation ethanol production is based on waste lignocellulosic biomass, sometimes even solving problems with waste management; therefore, it does not compete for raw materials used for food or feed production (Balat 2011; Sarkar et al. 2012). However, the effective use of plant biomass with a complex structure, mainly composed of cellulose, hemicellulose, and lignins, requires the use of an effective pretreatment method that would guarantee a reduction of crystalline areas in cellulose, as well as partial degradation of hemicellulose and lignins. At the same time, lignocellulose pretreatment should not lead to the formation of large amounts of fermentation inhibitors such as furfural and 5-hydroxymethylfurfural (5-HMF), which are products of sugar dehydration, nor produce phenolic compounds resulting from the degradation of lignins, e.g., vanillin, syringaldehyde, ferulic acid (Jonsson et al. 2013; Mood et al. 2013). Methods using elevated temperature and pressure associated with various chemical catalysts, i.e., diluted acids and bases, ionic liquids, or organic solvents, are the most popular and also the most effective plant biomass pretreatment techniques (Noureddini and Byun 2010; Li et al. 2010; Camasasca et al. 2015; Mou and Wu 2016; Smuga-Kogut et al. 2019). An effective way to create the necessary pressure is to heat the aqueous biomass suspension with microwaves (Bundhoo 2018; Mikulski et al. 2019; Mikulski and Kłosowski 2020a). Our and other authors’ studies clearly pointed to the usefulness of microwave pretreatment in a diluted sulfuric acid environment in the degradation of lignocellulose to simple sugars, which can then be used in bioconversion and biosynthesis processes (Ethaib et al. 2016; Germec et al. 2017; Amini et al. 2018; Mikulski et al. 2019; Mikulski and Kłosowski 2020a; Bhardwaj et al. 2020).

Alternatively, pretreatment of lignocellulosic biomass can be accomplished using compounds that partially remove lignins limiting the availability of cellulose fibers to cellulolytic enzymes. In the delignification process, hydrotropes, i.e., low molecular weight sodium or potassium salts of benzoic or arylsulfonic acid with a substituted alkyl group, are used. Similar to surfactants, they have an amphiphilic structure and reduce surface tension. However, unlike surfactants, they have smaller hydrophobic parts and do not self-aggregate into micelles (Dhapte and Mehta 2015; Devendra et al. 2016; Mikulski and Kłosowski 2020b). Compounds like sodium xylene sulfonate (NaXS) and sodium cumene sulfonate (NaCS) reduce the lignin concentration in plant biomass, depending on its origin, by ca. 38% in eucalyptus biomass (Mou and Wu 2016) and by ca. 47% in the mixture of beech and birch chips (Olsson et al. 2019). A very important aspect of using hydrotropes in the process of lignin extraction from lignocellulosic biomass is the possibility of recovering solutes (lignin) by diluting the hydrotrope solution, which can be reused after concentration (Devendra and Pandey 2016). Hydrotropic delignification is most effective at elevated temperatures and often has to be combined with subsequent baro-thermal treatment in a dilute acid environment. It leads not only to a decrease in lignin concentration but also to a high level of glucose released during enzymatic hydrolysis of cellulose (Devendra and Pandey 2016; Olsson et al. 2019; Mikulski and Kłosowski 2020b).

Effective pretreatment of lignocellulosic biomass, providing cellulose susceptible to hydrolysis, which at the same time does not burden the environment nor generate fermentation inhibitors, is a key element in the production of cellulosic ethanol. Currently, the search for new combinations of physicochemical methods of pretreatment focuses mainly on the use of environmentally friendly substances that can be used in the recirculation system under elevated pressure. Hydrotropes meet all of the abovementioned conditions. As shown in earlier studies, hydrotropes can be used at the initial stage to intensify subsequent processing, e.g., with diluted inorganic acids (Devendra and Pandey 2016; Mikulski and Kłosowski 2020b). The aim of the present study was to develop a one-step maize stillage biomass pretreatment method using NaCS together with microwave radiation to obtain a substrate susceptible to enzymatic hydrolysis of cellulose and maintain a low concentration of fermentation inhibitors. The research included selection of the conditions of microwave hydrotropic delignification (pressure and time of exposure as well as NaCS concentration), providing a maximum level of extraction of biomass components. The biomass with the highest level of lignin extraction was optimized for enzymatic hydrolysis and subsequent alcoholic fermentation to determine the degree of bioconversion of sugars to ethanol. The available literature on the subject lacks reports on hydrotropic delignification using microwave radiation. The authors believe that it is reasonable to combine these two pretreatment methods. The effectiveness of hydrotropes in the delignification process increases with increasing temperature and pressure, which is ensured by microwaves. The biomass obtained in such a process is susceptible to enzymatic hydrolysis. No additional catalysts, such as inorganic acids, are required.

## Material and methods

### Materials

The lignocellulosic raw material used in the study was centrifuged biomass of maize stillage supplied by an agricultural distillery (Gospodarstwo Rolne in Radzicz, Poland) which uses the classic baro-thermal technology in releasing the starch with Henze steamer. Before the experiments, the material was dried at 60 °C until constant weight, ground, and sieved through a 1-mm sieve. The raw material dry weight (DW) was 95.06 ± 0.15%. The content of cellulose, hemicellulose, and lignins was 34.15 ± 0.28%, 13.56 ± 0.48%, and 16.23 ± 0.67%, respectively. Analytical and HPLC (high-performance liquid chromatography)-grade reagents used in the study were supplied by Merck® (Darmstadt, Germany). The standards used in the chromatographic analyzes were HPLC-grade, supplied by Sigma-Aldrich® (St. Louis, MO, USA). Sodium cumene sulfonate (NaCS) was used as the hydrotrope (extraction solvents), in the form of a 40% v/v Stepanate® SCS 40 preparation supplied by the Stepan Company (Northfield, IL, USA).

### Enzymatic preparations

Cellic® CTec2 preparation (Novozymes, Franklinton, NC, USA) containing a complex of cellulolytic enzymes with 75 FPU (filter-paper units)/mL activity was used in the enzymatic hydrolysis of cellulose. The preparation was applied according to the manufacturer’s instructions at pH 5.5 and 50 °C. In the preparation of fermentation media, Viscozyme® L (Novozymes, Bagsvaerd, Denmark) was additionally used. This preparation exhibits endo-β-glucanase (xylanase, cellulase, hemicellulase) activity, 100 FBG/g, and hydrolyses β-(1,3)- or β-(1,4)-glycosidic linkages in β-D-glucans. It was used according to the manufacturer’s instructions at a dose of 8 FGB (beta glucanase units)/g DW, at pH 5.5 and 50 °C.

### Microorganism

The alcoholic fermentation process was carried out using an active preparation of dried yeast *Saccharomyces cerevisiae* strain Ethanol Red (Lesaffre Advanced Fermentations). Yeast was applied in the form of yeast milk (1.25 ± 0.12 × 10^9^ CFU/mL, cell viability 94.3 ± 0.5%) prepared by suspending 1 g of the preparation in 10 ml of sterile 0.9% v/v NaCl at 30 °C (according to the manufacturer’s instructions). The dose of yeast was 2 g of preparation per 1 L of cellulosic fermentation medium.

### Research stages

#### Selection of process conditions of microwave hydrotropic extraction

In the first stage of research, we examined the impact of the conditions of microwave hydrotropic extraction on biomass extractives, enzymatic hydrolysis of cellulose, and the composition of biomass. The effects of hydrotropic extraction under microwave conditions were evaluated for different pressure values (39, 78, 117 PSI), exposure time (10, 20, 30 min), and NaCS concentration (0, 10, 20% v/v), with constant microwave generator power at 300 W. The experiment included 27 variants of microwave hydrotropic extraction. The analysis began by placing 1 g DW (1.05 g) of a given type of biomass in a HP-500 plus Teflon container and adding 20 mL of extraction NaCS solution or water (5% biomass concentration). The extraction process was carried out in the MARS 5 microwave mineralizer (by CEM Corporation) to control the pressure conditions. After the extraction, the solution was cooled to ca. 60 °C and filtered through a polyamide filter membrane with a pore size of 50 μm. The biomass was then rinsed with 150 mL of hot distilled water to remove residual extraction solution, and dried to constant weight (DW) at 130 °C using a moisture analyzer. The biomass extractives were calculated based on the difference in sample weight before and after hydrotropic extraction (Devendra and Pandey 2016). The analysis was performed in 9 replications to obtain enough biomass necessary to calculate the biomass extractives and to obtain the raw material for the analysis of lignocellulose composition and to carry out the enzymatic hydrolysis process. After microwave extraction, the biomass was dried up and then used to determine the content of cellulose, hemicellulose, and lignins. The analysis of the impact of the conditions of microwave hydrotropic extraction on the efficiency of enzymatic hydrolysis of cellulose began with placing 1 g DW (1.05 g) of the biomass after extraction in a 250-mL conical flask and adding 25 mL 0.05 M acetate buffer pH 5.5 (4% biomass concentration). The solution was then placed in a shaking water bath at 50 °C. 5 FPU Cellic® CTec2 cellulolyte preparation (65 μL) was added and shaken for 72 h. Glucose concentration was measured every 24 h by HPLC. Analysis of the biomass composition and the impact of the hydrotropic microwave extraction conditions on the amount of glucose obtained from the biomass after the enzymatic hydrolysis of cellulose were carried out in triplicate.

#### Selection of conditions for cellulose enzymatic hydrolysis

Optimization of the enzymatic hydrolysis process of cellulose was carried out on biomass after microwave hydrotropic extraction under the following conditions: pressure 117 PSI, extraction time 30 min, NaCS concentration 20% v/v, microwave generator power 300 W. Optimization of hydrolysis conditions included selection of biomass concentration (4, 8, 16% w/v) and enzyme amount (1, 2, 4 FPU/g DW biomass) in a 72-h process. The analysis began by placing 1, 2, or 4 g DW (1 g DW = 1.05 g) biomass after microwave hydrotropic extraction in 250-mL conical flasks and adding 25 mL 0.05 M acetate buffer pH 5.5. Then, the solution was placed in a shaking water bath at 50 °C. Cellic® CTec2 cellulolytic preparation was added (assuming that 13 μL corresponded to 1 FPU/g DW). The hydrolysis lasted 72 h. Glucose concentration was measured every 24 h by HPLC. All analyzes were performed in triplicate. Based on glucose concentration, the yield of cellulose hydrolysis was determined as follows:

1 $$ \mathrm{Yield}\ \mathrm{of}\ \mathrm{hydrolysis}\ \left[\%\right]=\frac{C_{\mathrm{Glu}}}{1.111\times {C}_{\mathrm{C}\mathrm{el}}\times {\mathrm{C}}_{\mathrm{Biom}}}\times 100 $$

where *C*_Glu_ is the concentration of glucose (grams per liter) in the sample, *C*_Cel_ is the cellulose content in biomass (per 1 gram of dry weight), and *C*_Biom_ is the initial biomass content (grams per liter) (Rana et al. 2014).

#### Assessment of the suitability of microwave hydrotropic extraction in the production of ethanol from maize stillage biomass

Alcoholic fermentation was carried out using stillage biomass after microwave hydrotropic extraction under the conditions selected in the first stage of the study (pressure 117 PSI, extraction time 30 min, NaCS concentration 20% v/v, microwave generator power 300 W). The biomass after hydrotropic treatment was subjected to enzymatic hydrolysis using Cellic® CTec2 preparation under the conditions selected in the second stage of the study, ensuring the highest glucose concentration during a 24-h process, i.e., biomass concentration 16% w/v and 4 FPU/g DW. In subsequent research variants, an additional Viscozyme® L preparation was applied for a more complete use of polysaccharide components in the fermentation process. Alcoholic fermentation was carried out in three variants (Table 1). As for the fermentation environment, we used acetate buffer, to provide optimal conditions for enzymatic hydrolysis and fermentation, or tap water to simulate the conditions of the technological process. Preparation of the fermentation medium was started by placing 8 g DW of stillage biomass in a 250-mL conical flask and adding 50 mL of the appropriate solvent (depending on the test variant). When water was used, the pH was adjusted to 5.5 with 0.1 M NaOH and 0.1 M HCl. Then, the Cellic® CTec2 enzyme preparation was added, 4 FPU/g DW. Viscozyme® L, 8 FGB/g DW, was additionally applied in selected experiments (Table 1). Enzymatic hydrolysis was carried out in a shaking water bath at 80 rpm and 50 °C for 24 h. After the hydrolysis, the solution was cooled down to 35 °C and inoculated with yeast milk. Then, the flask was sealed with a fermentation tube filled with glycerin and incubated at 35 °C for 72 h. The composition of the fermentation medium was examined by HPLC before fermentation and every 24 h of the process. The analysis included determining the concentration of sugars, glycerol, organic acids, ethanol, phenolic compounds, 5-HMF, and furfural. Fermentation tests were carried out in triplicate. Fermentation yield after 72 h of the process was calculated based on the concentration of ethanol and sugars in the fermentation medium according to the formula:

2 $$ \mathrm{Fermentation}\ \mathrm{yield}\ \left[\%\right]=\frac{E}{\mathrm{SC}\times 0.511}\times 100 $$

where *E* is the concentration of ethanol (grams per liter) after 72 h of the process, SC is the initial content of sugars (glucose, xylose/galactose, in grams per liter), and 0.511 is the value representing the theoretical ethanol yield from polysaccharides (Berłowska et al. 2016).

**Table 1:** Characteristics of fermentation media prepared during the research

Research variant	Biomass pretreatment conditions	Solvent	pH	Enzyme preparation
MHF 1	Pressure 117 PSI, extraction time 30 min, NaCS concentration 20% v/v, microwave generator power 300 W	0.05 M acetate buffer	5.5	Cellic® CTec2
MHF 2		0.05 M acetate buffer	5.5	Cellic® CTec2 Viscozyme® L
MHF 3		Water	5.5	Cellic® CTec2 Viscozyme® L

### Analytical methods

#### Characterization of native and pretreated distillery stillages

Determination of cellulose, hemicellulose, and lignins was performed using the FOSS Fibertec 8000® system. The analysis was based on the extraction and weighing of neutral detergent fiber (NDF), acid detergent fiber (ADF), and acid detergent lignin (ADL). Prior to determining the concentration of structural polysaccharides, the starch was removed by enzymatic hydrolysis with α-amylase. Measurements were carried out in accordance with the device manufacturer’s methodology, and according to ISO 13906: 2008 and ISO 16472: 2006.

#### Determination of carbohydrates, acetic acid, glycerol, and ethanol in fermentation medium

The content of sugars (glucose, galactose and xylose, arabinose), acetic acid, glycerol, and ethanol in the samples after enzymatic hydrolysis of cellulose and in fermentation media was analyzed by high-performance liquid chromatography (HPLC-RID). Before analysis, the samples were diluted 5-fold in the mobile phase, i.e., 5 mM H_2_SO_4_, and filtered through a 0.45-μm membrane filter. The analysis was carried out using an Agilent Technologies® model 1260 chromatograph equipped with a refractometric detector. The separation was performed on a Hi-Plex H column (Agilent Technologies®) equipped with a dedicated guard column, with a mobile phase flow of 0.6 mL/min at 60 °C. Quantitative analysis was performed using external standards (ESTD). Because Hi-Plex H column does not allow for effective separation of xylose and galactose peaks, the concentration of these sugars is given as their sum.

#### Determination of 5-HMF, furfural, and phenolic compounds as lignin degradation products

Phenolic compounds, i.e., vanillin, 4-hydroxybenzoic acid, syringaldehyde, trans-ferulic acid (as lignin degradation products) as well as 5-HMF, and furfural, were also determined in fermentation media. The analysis was performed using the Agilent Technologies® HPLC system, model 1260, equipped with a diode detector (HPLC-DAD). Chromatographic separation was carried out on a ZORBAX Eclipse Plus C18 (4.6 × 100 mm, 3.5 μm) column (Agilent Technologies®) using 0.3% acetic acid (70%) and methanol (30%) as mobile phase, at a flow rate of 0.5 mL/min and 30 °C. 5-HMF, furfural, 4-hydroxybenzoic acid, and vanillin were detected at 280 nm. Syringaldehyde and trans-ferulic acid were detected at 320 nm (Cho et al. 2009). Quantitative analysis was performed using ESTD. The concentration of phenolic lignin degradation products, i.e., syringaldehyde, trans-ferulic acid, 4-hydroxybenzoic acid, and vanillin, was analyzed in the range from 15 to 300 mg/L. The concentration of 5-HMF and furfural was analyzed in the range from 40 to 200 mg/L. The detection limits for the individual compounds were as follows: syringaldehyde LOD < 0.18 mg/L, trans-ferulic acid LOD < 0.15 mg/L, vanillin LOD < 0.32 mg/L, 4-hydroxybenzoic acid LOD < 0.12 mg/L, 5-HMF LOD < 1.40 mg/L, furfural LOD < 2.75 mg/L.

### Statistical analysis

All laboratory analyzes were performed in triplicate. Statistical analysis was carried out using the Statistica software ver. 12 (analysis of variance, determination of SD). ANOVA test and HSD Tukey’s test were applied at the significance level of α < 0.05.

## Results

### Selection of conditions for microwave hydrotropic extraction

**Fig. 1:**
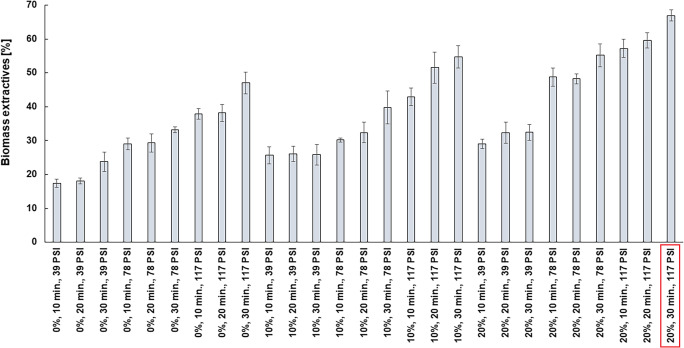
The effect of NaCS concentration, pretreatment time, and pressure on biomass extractives after microwave-assisted hydrotropic pretreatment of maize distillery stillage

We analyzed the effect of various combinations of conditions of microwave hydrotropic treatment, i.e., NaCS concentration (0, 10, 20% v/v), exposure time (10, 20, 30 min), and pressure (39, 78, 117 PSI) on the extraction of biomass components, changes in biomass composition (cellulose, hemicellulose, and lignin content), and the amount of glucose released from pre-processed biomass as after hydrolysis with cellulases. The experiments showed that effective extraction of components of maize stillage biomass was possible even when only water was used as the solvent (0% v/v NaCS). This confirms the effectiveness of microwave treatment in the extraction processes. Lignocellulosic biomass extractives increased with exposure time and increasing pressure (Fig. 1). Higher pressure during the extraction was associated with higher temperature, which promoted dissolution of solid components. The extraction of biomass components also increased with increasing NaCS concentration, as a result of a decrease in the surface tension of the solution used. The highest biomass extractives, 67.00 ± 1.68%, were observed for the highest NaCS concentration (20% v/v), 30-min exposure time, and 117 PSI (Fig. 1). Biomass extractives in this extraction variant were more than 12% higher compared to 10% v/v NaCS extraction and more than 20% higher than with water-only extraction, with the remaining process parameters unchanged (Fig. 1).

**Fig. 2:**
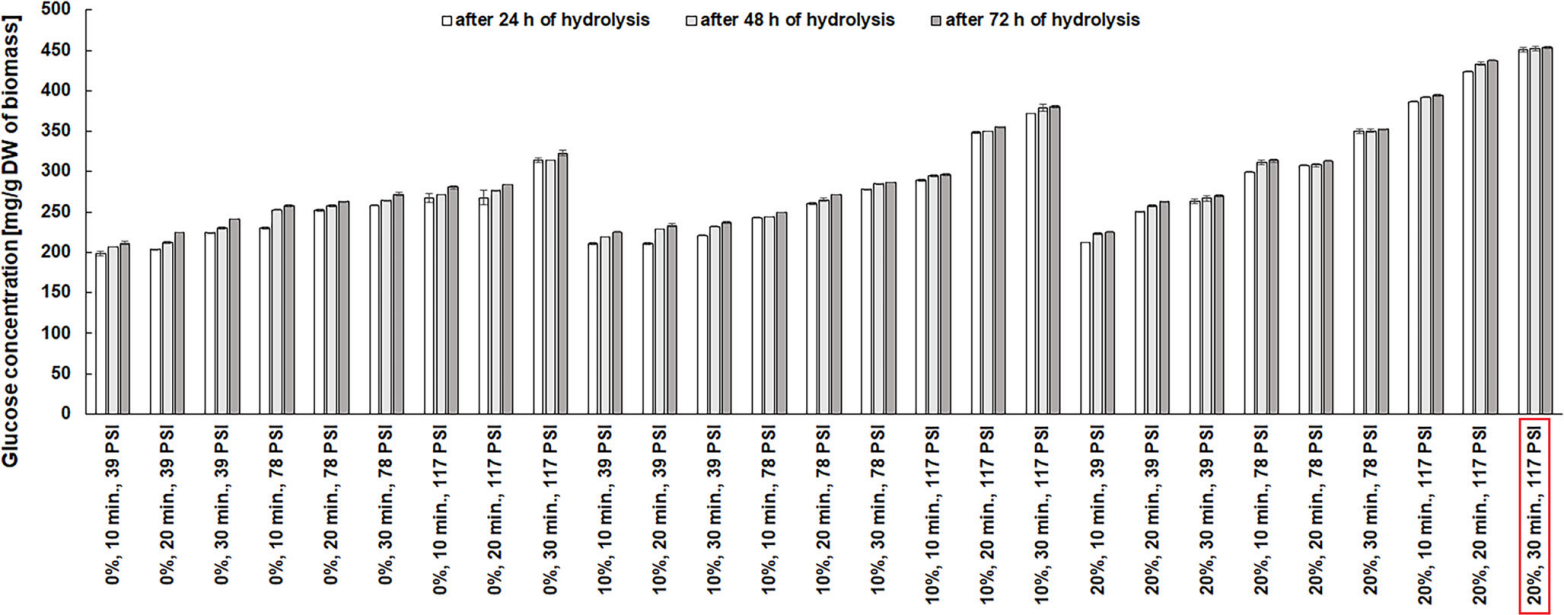
The effect of NaCS concentration, pretreatment time, and pressure on glucose concentration obtained by the enzymatic hydrolysis of cellulose after microwave-assisted hydrotropic pretreatment of maize distillery stillage

The microwave pretreatment with the use of a hydrotrope carried out in various process conditions caused changes in the composition of maize stillage biomass (Fig. 2). The use of water as an extraction solution during microwave treatment resulted in a partial extraction of organic matter from biomass. Water as the solvent did not have a major impact on the changes in cellulose concentration in biomass (average value around 37%), but it did lower hemicellulose levels and caused an increase in lignin concentration as extraction progressed, which was due to the non-polar structure of lignins (Table 2). The increase in lignin concentration was associated with the research methodology and resulted from the use of a constant amount of biomass after microwave extraction to analyze the biomass composition. The extraction with 10% v/v NaCS resulted in changes in the biomass content. The concentration of cellulose increased and the content of hemicellulose and lignins decreased as extraction progressed and pressure increased (Table 2). After 30 min of extraction with 20% v/v NaCS at 117 PSI, this effect was enhanced: the concentration of cellulose, hemicellulose and lignins in biomass was 56.70 ± 0.26%, 1.55 ± 0.99%, and 14.97 ± 0.32%, respectively (Table 2). The maximum reduction of lignin concentration in these conditions (20% v/v NaCS) was about 44% compared to the process using only water, with the remaining process parameters unchanged. The results leave no doubt as to the suitability of microwave radiation combined with the use of NaCS for maize stillage biomass delignification. The delignification effect is visible despite the high biomass extractives (about 67%). Because a constant amount of biomass is used to determine the components of lignocellulose, the effects of the lignin extraction process are somewhat covered up.

**Table 2:** The effect of NaCS concentration, pretreatment time, and pressure on biomass composition after microwave-assisted hydrotropic pretreatment of maize distillery stillage

Pretreatment pressure (PSI)	Pretreatment time (min)	NaCS concentration (%)	Biomass component (% of dry weight)			NaCS concentration (%)	Biomass component (% of dry weight)			NaCS concentration (%)	Biomass component (% of dry weight)		
			Cellulose	Hemicellulose	Lignin		Cellulose	Hemicellulose	Lignin		Cellulose	Hemicellulose	Lignin
39	10	0	36.81 ± 0.79	14.63 ± 0.49	18.27 ± 0.47	10	39.48 ± 0.73	2.64 ± 0.96	25.81 ± 0.06	20	50.52 ± 0.45	2.44 ± 0.33	18.53 ± 1.27
39	20	0	35.05 ± 0.32	13.04 ± 0.42	21.97 ± 0.84	10	42.76 ± 0.45	7.93 ± 1.24	21.56 ± 1.90	20	51.57 ± 0.28	6.59 ± 0.24	18.16 ± 0.15
39	30	0	34.33 ± 1.45	10.50 ± 0.76	24.07 ± 1.59	10	42.02 ± 0.56	7.07 ± 1.77	20.41 ± 0.20	20	53.36 ± 1.41	5.60 ± 0.18	16.49 ± 1.31
78	10	0	38.36 ± 0.24	12.51 ± 0.01	19.15 ± 0.33	10	38.49 ± 0.58	5.90 ± 0.31	24.24 ± 0.47	20	49.65 ± 0.06	3.07 ± 0.05	18.57 ± 0.79
78	20	0	35.38 ± 0.79	10.43 ± 0.79	24.95 ± 0.32	10	40.89 ± 0.16	6.42 ± 0.11	21.10 ± 0.26	20	51.26 ± 0.00	4.39 ± 0.21	18.52 ± 0.11
78	30	0	38.26 ± 0.91	4.48 ± 1.04	25.85 ± 0.72	10	42.47 ± 0.05	7.02 ± 0.60	20.67 ± 0.15	20	54.95 ± 0.65	3.08 ± 0.01	16.97 ± 0.74
117	10	0	38.29 ± 0.98	8.27 ± 0.47	20.74 ± 0.75	10	42.02 ± 2.02	6.28 ± 0.18	21.82 ± 0.48	20	48.54 ± 0.01	5.18 ± 0.05	18.01 ± 0.23
117	20	0	38.01 ± 0.94	7.45 ± 0.41	23.72 ± 0.47	10	42.52 ± 0.37	2.37 ± 0.74	20.68 ± 0.16	20	55.24 ± 0.11	1.23 ± 0.34	17.10 ± 0.06
117	30	0	38.10 ± 0.61	6.24 ± 0.56	26.48 ± 0.22	10	46.78 ± 0.60	1.87 ± 0.61	20.26 ± 0.06	20	56.70 ± 0.26	2.55 ± 0.99	14.97 ± 0.32

Biomass samples after hydrotropic microwave delignification were also subjected to enzymatic hydrolysis using a cellulolytic enzyme. The purpose of this research stage was to determine the effect of various process conditions of microwave hydrotropic delignification on glucose concentration after enzymatic hydrolysis of cellulose. Studies confirmed a high efficiency of hydrolysis of cellulose in maize stillage biomass after microwave hydrotropic delignification. The highest glucose concentration in the hydrolyzate was over 450 mg/g DW. This concentration was achieved after microwave treatment with NaCS under conditions providing the highest biomass reduction and the highest cellulose concentration, i.e., at 20% v/v NaCS, 117 PSI, and 30 -min exposure time (Fig. 2).

### Optimization of enzymatic hydrolysis of cellulose in biomass after microwave hydrotropic delignification

**Fig. 3:**
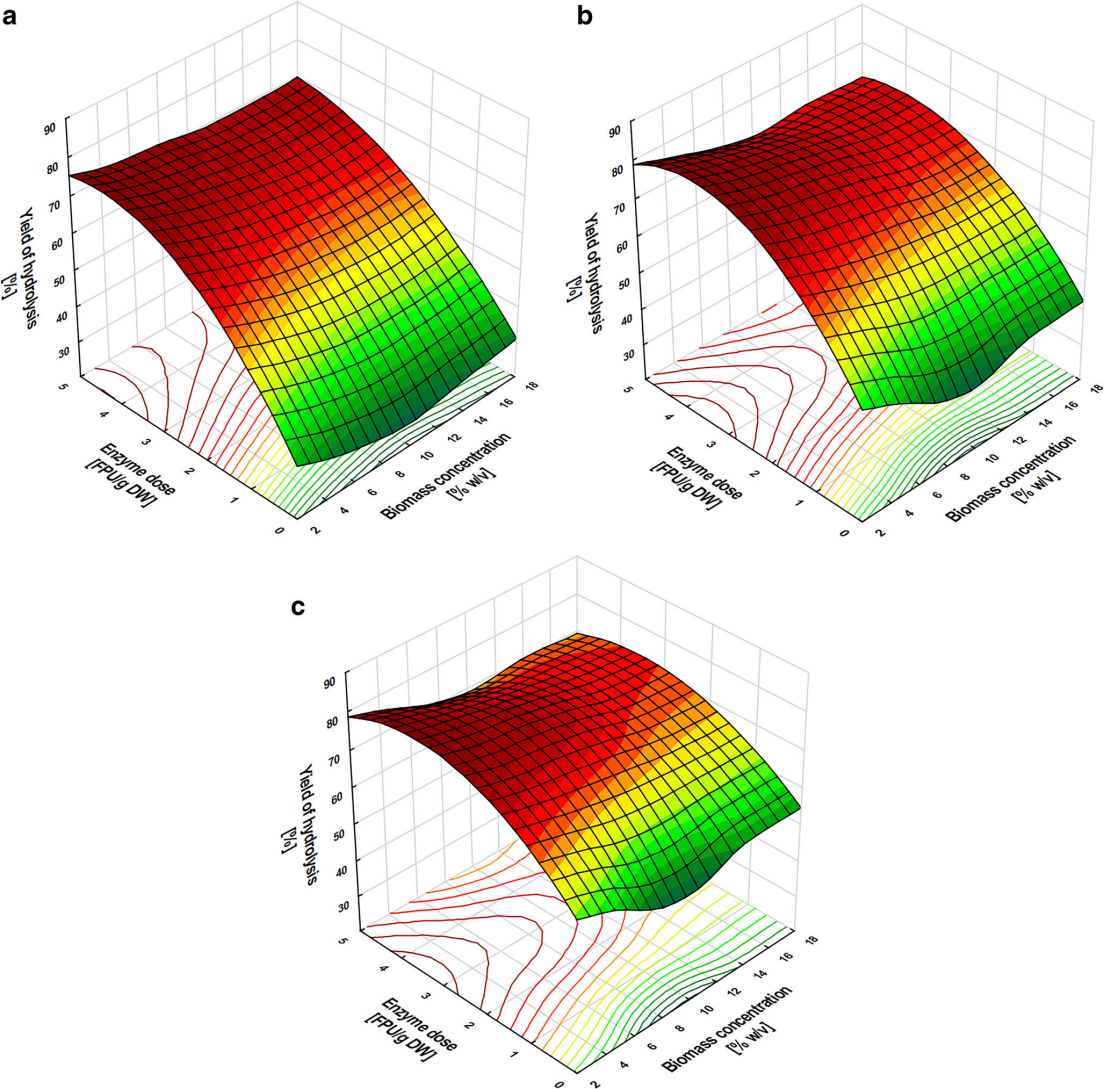
Response surface plot showing the effect of the interaction between maize stillage biomass concentration and enzyme dose on hydrolysis yield after 24 (**a**), 48 (**b**), and 72 (**c**) h of enzymatic hydrolysis

The next stage of the study after choosing the parameters of microwave hydrotropic delignification was the optimization of conditions for cellulose enzymatic hydrolysis. To this end, maize stillage biomass previously subjected to microwave hydrotropic treatment with 20% v/v NaCS at 117 PSI for 30 min was hydrolyzed using a cellulase preparation in acetate buffer pH 5.5. The hydrolysis process was analyzed for three biomass concentrations (4, 8, 16% w/v) and three enzyme dose levels (1-, 2-, and 4-FPU/g DW biomass). All experiments were run for 72 h. The highest cellulose hydrolysis yield, c. a. 80%, was obtained for 4% w/v biomass concentration at the highest enzyme dose (4-FPU/g biomass) after 48 h of the process (Fig. 3). Importantly, there were no statistically significant differences in cellulose hydrolysis yield between different biomass concentrations (4, 8, 16% w/v) at a given hour using a given dose of enzyme. A clear increase in cellulose hydrolysis yield in subsequent hours of the process was observed only for enzyme doses of 1- and 2-FPU/g biomass. The yield difference between the 24th and 72nd hour of hydrolysis at a dose of 1-FPU/g biomass ranged from about 30% for 4% w/v biomass concentration to about 20% for 16% w/v (Fig. 3). For the enzyme dose increased to 2-FPU/g biomass, the yield difference between 24th and 72nd hour of hydrolysis was around 20% regardless of the biomass concentration used. At the highest enzyme dose, 4-FPU/g biomass, the yield of cellulose hydrolysis after 72 h was only 8% higher than after 24 h of the process, regardless of the biomass concentration in the medium (Fig. 3). The regression coefficient (*R*^2^) was 95.87%, which indicated that the model was suitable for mapping the actual relationships between selected variables of enzymatic reactions. Conditions that ensured high glucose level after 24 h of hydrolysis, i.e., 16% w/v biomass concentration and enzyme dose of 4-FPU/g biomass, were selected for the next stage of the study.

### Evaluation of the suitability of hydrolyzate from maize stillage after microwave hydrotropic treatment and enzymatic hydrolysis for the production of cellulosic ethanol

**Fig. 4:**
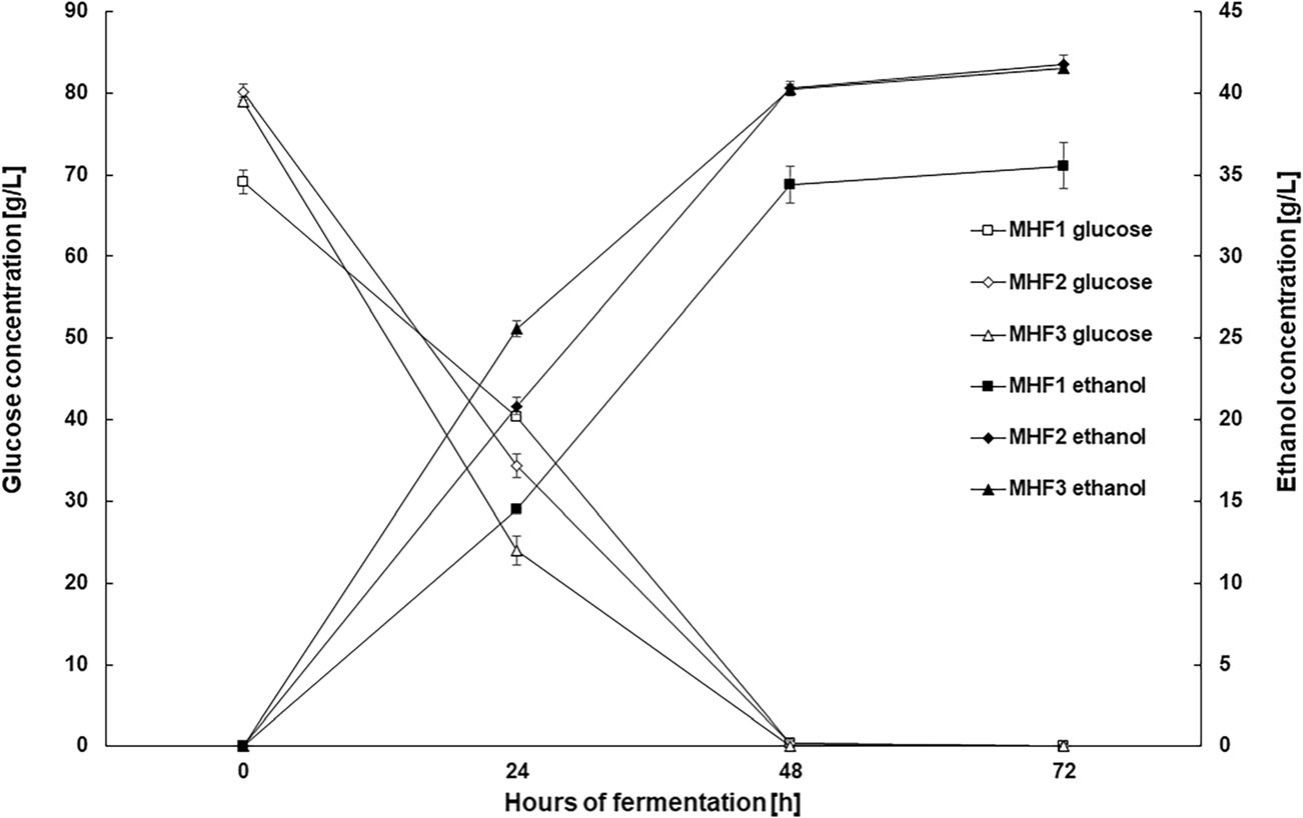
Changes in the concentration of glucose (open tags) and ethanol (closed tags) during alcoholic fermentation

**Fig. 5:**
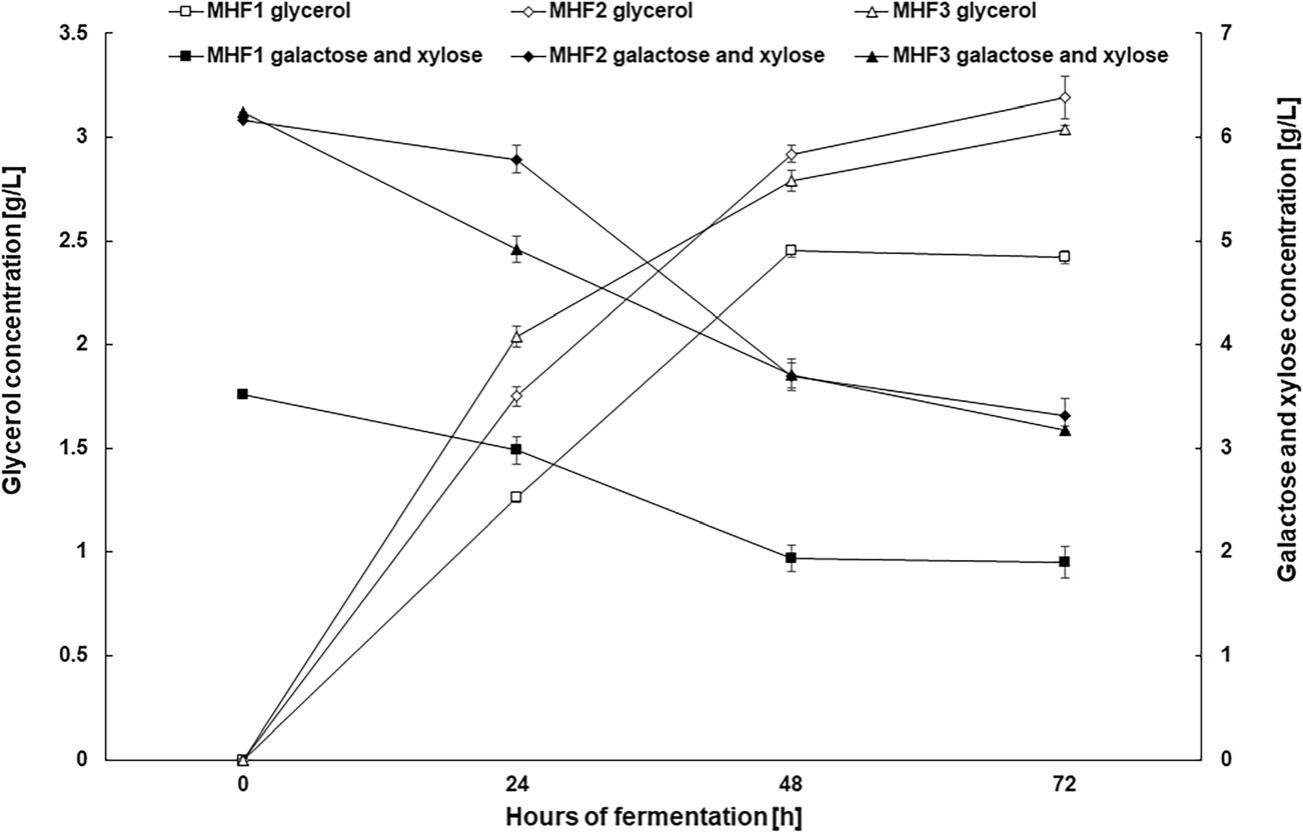
Changes in the concentration of glycerol (open tags), galactose, and xylose (closed tags) during alcoholic fermentation

The main objective at this stage of the study was to assess the suitability of maize stillage subjected to microwave hydrotropic treatment for ethanol fermentation. In order to obtain the highest initial concentration of fermentable sugars, two different solvents (acetate buffer and water) as well as different enzyme preparations were analyzed. All fermentation media at this stage of the study were prepared using the previously selected, most effective combination of pretreatment parameters: 117 PSI, extraction time 30 min, and 20% v/v NaCS. The hydrotropic microwave treatment with NaCS proved to be a very effective way of preparing maize stillage biomass for fermentation. It is worth noting that the proposed simplified pretreatment, involving only NaCS and microwaves (without additional acid treatment), allows to obtain a fermentation medium containing up to 80 g of glucose per liter, without condensing. This result was achieved after enzymatic hydrolysis of structural polysaccharides (such as cellulose and hemicellulose) present in 160 g/L of biomass (Fig. 4). Eliminating the need to condense the substrate to obtain a high concentration of fermentable sugars is an important achievement of the proposed method. It suggests that microwave hydrotrope can improve the economics of cellulosic ethanol production. In the simplest experimental variant (MHF 1), only cellulolytic enzymes from Cellic® CTec2 preparation were used to hydrolyze cellulose after pretreatment of biomass. In order to create optimal conditions for these biocatalysts, acetate buffer pH 5.5 was used as the solvent. In other experimental variants (MHF 2 and 3), in addition to Cellic® CTec2, hemicellulose-degrading enzymes were used to increase the amount fermentable sugars, i.e., glucose and galactose. In these variants, acetate buffer pH 5.5 (MHF 2) or water pH 5.5 (MHF 3) was used as the solvent (Table 1). The lowest initial concentrations of glucose, ca. 69 g/L, as well as galactose and xylose, ca. 3.5 g/L, were observed in the MHF 1 variant, because in this experiment, only cellulolytic enzyme was applied (Table 1, Figs. 4 and 5). When additional hemicellulose-degrading enzymes were used, a higher glucose concentration (ca. 79 g/L) was obtained, irrespective of the solvent used (buffer or water); galactose and xylose level also increased to ca. 6 g/L (Figs. 4 and 5). Arabinose was not found in any of the fermentation media. It is worth emphasizing that fermentation media obtained after microwave hydrotropic treatment of maize stillage biomass contained 5-HMF, furfural, and lignin degradation products such as syringaldehyde, trans-ferulic acid, vanillin, and 4-hydroxybenzoic acid below the detection limits. The initial phase of fermentation in such media was not impeded, as observed in media with inhibitors. The undisturbed course of the initial fermentation phase resulted in complete bioconversion of glucose to ethanol after 48 h of the process, in each experiment (Fig. 4). In experiments with Viscozyme® L (MHF 2 and 3), between 24 and 48 h of fermentation, only galactose was fermented, which led to a decrease in the sum of this sugar and xylose by ca. 0.5 g/L (Fig. 5). Ethanol Red strain does not exhibit the ability to assimilate xylose, which leads to the accumulation of small amounts of this sugar in the fermented medium (up to ca. 3 g/L). This amount of xylose converted to ethanol would have little effect on ethanol yield (assuming a conversion rate of 0.511). However, the solution after alcohol distillation can be used in biosynthesis processes with microorganisms capable of xylose conversion. Data collected during fermentation experiments suggest that it is possible to achieve relatively high ethanol concentrations in lignocellulosic media, e.g., 35.54 ± 1.42 g/L for MHF 1 and c.a. 41.5 g/L for MHF 2 and 3 (Fig. 4). Statistical analysis showed no significant differences between the initial glucose, galactose, and xylose concentrations and the final ethanol concentration in MHF variants 2 and 3. The absence of fermentation inhibitors in the media combined with the high initial concentration of fermenting sugars resulted in a high concentration of ethanol and high fermentation yield. After 72 h of the process, the ethanol yield in relation to the theoretical one was about 95%, in all variants (Table 3). High yeast activity also exerted an effect on glycerol concentration in fermentation media (Fig. 5). This work demonstrated that maize stillage biomass after initial microwave hydrotropic treatment and enzymatic hydrolysis can be used to produce second-generation ethanol. It is worth noting that achieving a high attenuation level did not require any supplementation with mineral substances or additional source of nitrogen, which otherwise would also affect the profitability of the process.

**Table 3:** Alcoholic fermentation yield relative to theoretical yield after 72 h of the process

Research variant	Yield of alcoholic fermentation (% of theoretical yield of glucose, galactose, and xylose) after 72 h
MHF 1	95.75a ± 0.86
MHF 2	94.69a ± 0.61
MHF 3	95.33a ± 0.57

The mean values given in columns with different letter index are significantly different (*α* < 0.05)

## Discussion

Against the background of the current state of knowledge, the results showing effects of the combined application of microwaves and hydrotropes in plant biomass extraction are an element of scientific novelty. The positive influence of high temperature and elevated hydrotrope concentration on the extraction process was reported in previous studies. Microwave-assisted hydrotropic delignification of maize stillage made it possible to extract components of biomass that are sparingly soluble in water solutions, such as lignins and raw oils. The addition of hydrotropes reduced the surface tension and increased the solubility of these compounds. In addition, the biomass extractives also contained proteins, glycerol (which is a by-product of alcoholic fermentation), and minerals (including silica). The maize stillage biomass did not contain any residual starch (Noureddini and Byun 2010). Increased temperature disrupts bonds in lignocellulosic biomass. This effect, in combination with the amphiphilic structure of hydrotropes and their ability to increase the solubility of organic compounds in aqueous solutions, increases the extraction (Balasubramanian et al. 1989). In our earlier study, we also used the maize stillage biomass as a source of lignocellulose, but the maximum extractives were only 25.56 ± 1.25% at 131 °C (40.15 PSI) with 20% v/v NaCS (Mikulski and Kłosowski 2020b). The maximum extraction of biomass components from rice straw using 20% v/v NaCS at 131 °C was 31.07 ± 2.47% (Devendra and Pandey 2016). In the present study, a much higher level of extraction of biomass components using NaCS was achieved due to the combined use of microwave radiation. The usefulness of microwaves in the extraction of biomass components was also confirmed for the NaOH solution. After vine shoot processing with microwave radiation and 3.1% w/v NaOH at 125 °C, biomass extractives increased to 54% (Dávila et al. 2018). Other authors also confirmed the dependence of extraction on the type and concentration of the solution used for pretreatment. In the microwave-assisted treatment of dragon fruit, biomass extractives increased from ca. 4 to 11% and from ca. 7 to over 12% with increasing concentration of sulfuric acid (from 0.01 to 0.1 N) and sodium hydroxide (from 0.01 to 0.1 N), respectively (Ethaib et al. 2016). A high level of extraction of vine shoot biomass components (ca. 53%) was also observed when microwave radiation was used along with NaOH solution under optimized process conditions, i.e., temperature (150 °C), time (30 min), and NaOH concentration (3.1% w/v) (Dávila et al. 2019).

To our knowledge, no reports describing microwave delignification using hydrotropes are available in the literature. However, many studies have confirmed that hydrotropes are effective in the extraction of lignins from plant biomass. Sodium xylene sulfonate (NaXS) used in the delignification process reduced lignin concentration by ca. 28% in eucalyptus biomass, by ca. 80% in sugar cane bagasse, by ca. 12% in wheat straw, and by ca. 47% in mixed birch and beech chips. Note that achieving this level of delignification required 30% NaXS applied at 110 °C (20 PSI) in sugar cane bagasse treatment or > 150 °C (> 68 PSI) in the treatment of other materials (wheat straw, a mixture of birch and beech chips, eucalyptus biomass); the exposure time was from1 to 8 h (Bundhoo 2018; Olsson et al. 2019; Ansari and Gaikar 2014; Qi et al. 2019). Studies demonstrated that NaCS could remove ca. 7 to 18% of lignins from distillery stillage biomass, ca. 30% from cotton stalks, and ca. 52% from rice straw. Such reduction of lignin concentration was achieved for 20% v/v NaCS, at 131 °C in the extraction lasting at least 1 h (Devendra and Pandey 2016; Karthyani et al. 2017; Mikulski and Kłosowski 2020b). As a result of the combined use of microwave radiation with NaCS, as much as 44% reduction in lignin concentration was achieved in a very short time (at most 30 min); none of the previous studies reported such a large reduction in such a short time. The usefulness of microwaves in the delignification process was also confirmed by other studies. For pretreatment in combination with microwave radiation, sulfuric acid, ionic liquids, or basic salts with orthophosphoric acid are most often used. Microwaves together with 0.5% v/v sulfuric acid reduced the concentration of lignins in aloe biomass by up to 66% (Rajeswari et al. 2020). The combined action of ionic liquids together with microwave radiation at 130 °C resulted in a nearly 80% reduction in lignin concentration in *Miscanthus* biomass and birch wood (Kohli et al. 2020). Pretreatment with microwaves and basic salts in orthophosphoric acid reduced the lignin concentration in rice straw by only 12 to 20% (Bhardwaj et al. 2020). Similar to our study, it was found that as a result of extraction of organic substances from plant biomass, the concentration of cellulose increased.

Studies confirmed a high efficiency of hydrolysis of cellulose in maize stillage biomass after microwave hydrotropic delignification. Obtaining such a high (450 mg/g DW) glucose concentration per gram of biomass after pretreatment was not possible in our previous studies on the same raw material, which was subjected to baro-thermal treatment combined with diluted sulfuric acid (Mikulski and Kłosowski 2018). In that experiment, a maximum of about 225 mg glucose per g of biomass was achieved. However, the use of dilute sulfuric acid together with microwave radiation resulted in ca. 270 mg of glucose per gram of biomass after cellulose hydrolysis (Mikulski et al. 2019). The maximum glucose concentration obtained after enzymatic hydrolysis of 1 g of maize stillage subjected to microwave hydrotropic delignification was also more than three times higher compared to that without pretreatment. Glucose concentration after enzymatic hydrolysis of untreated stillage cellulose did not exceed 141.6 ± 3.6 g/g of biomass (Mikulski and Kłosowski 2018). The susceptibility of biomass after hydrotropic treatment to enzymatic hydrolysis using cellulases was also demonstrated in studies on eucalyptus biomass. In eucalyptus biomass pretreated with NaXS, the conversion of cellulose to glucose was as high as about 80% (Mou and Wu 2016). In our previous studies on the same raw material after baro-thermal treatment in a dilute sulfuric acid environment, the maximum yield of cellulose hydrolysis was 70% (it was lower by ca. 10% compared to this work), but only after 72 h of the process (Mikulski and Kłosowski 2018). In studies using corn stover, the highest cellulose hydrolysis yields were reported only at the highest enzyme concentration (40 mg protein per 1 g of glucan) and the highest biomass concentration (20% w/v). At lower enzyme concentrations, a significant decrease in hydrolysis yield was observed, even by 35% (Rana et al. 2014). Also, authors using rice straw as lignocellulosic raw material pointed out that obtaining the highest hydrolysis yield required precise optimization of process parameters. Properly selected enzyme and biomass concentrations resulted in up to 86% cellulose hydrolysis yield (Jeya et al. 2009).

A review of the literature indicates the innovative nature of the proposed technological solution to use microwave radiation along with hydrotropes to process lignocellulosic biomass in ethanol production. Only a few studies attempted to use plant biomass after hydrotropic treatment at elevated temperature and pressure in the production of cellulosic ethanol. However, no previous study has achieved such a high concentration of fermentable sugars in the culture medium as in the present work. For example, the fermentation medium prepared from cotton stalks after hydrotropic treatment contained only 5.15 g of reducing sugars per liter (Karthyani et al. 2017). The use of NaCS and NaXS in the processing of rice straw delivered 19.74 g and 10.22 g of glucose per liter of fermentation medium, respectively (Devendra and Pandey 2016). In previous studies, we used NaCS to pretreat maize stillage biomass. The process was carried out at 131 °C for 1 h. The final glucose concentration in the fermentation medium was about 63 g/L, but only after condensing (Mikulski and Kłosowski 2020b). Other authors reported that rice straw after hydrotropic treatment can be a source of cellulose susceptible to enzymatic hydrolysis and could provide an ethanol yield of 73 or 78% relative to the theoretical yield depending on the hydrotrope used. They pointed out that effective removal of hydrotropes from biomass is important, because residues of these compounds at higher concentrations might adversely affect the yeast metabolism (Devendra and Pandey 2016). Hydrotropic treatment can also be useful in the preparation of wheat straw in the butanol biosynthesis process: the use of NaXS provided ca. 23 g/L glucose and, after conversion, over 9 g of butanol per liter (Qi et al. 2019).

This work was aimed at developing a new method of maize stillage biomass pretreatment. The method is based on microwave hydrotropic treatment with NaCS and can be used in the production of second-generation ethanol. It was demonstrated that the proposed method was an effective way to delignify maize stillage biomass, which reduced lignin concentration by ca. 44%. The stillage biomass after microwave treatment with NaCS was a good raw material for the production of second-generation ethanol because it exhibited a high susceptibility to hydrolysis using cellulolytic enzymes. The hydrolysis yield reached 80%. It is worth noting that the prepared media had a high glucose content of 80 g/L (without concentration) and did not contain fermentation inhibitors such as 5-HMF, furfural, or phenolic compounds. Elimination of fermentation inhibitors increased the rate of the fermentation process and led to a very high yield in relation to theoretical one, at the level of 95%. Such a yield was obtained without supplementation with nutrients. Therefore, microwave hydrotropic treatment using NaCS is an effective way of preparing waste stillage biomass for the production of cellulosic ethanol and can contribute to the integration of the production of first and second-generation ethanol.

## Data Availability

Not applicable. The article pre-print can be found in the repository at the link (https://www.researchsquare.com/article/rs-36466/v1).
